# Management of *Listeria monocytogenes* on Surfaces via Relative Air Humidity: Key Role of Cell Envelope

**DOI:** 10.3390/foods10092002

**Published:** 2021-08-26

**Authors:** Fiona Zoz, Stéphane Guyot, Cosette Grandvalet, Mélanie Ragon, Eric Lesniewska, Sébastien Dupont, Olivier Firmesse, Brigitte Carpentier, Laurent Beney

**Affiliations:** 1 Univ. Bourgogne Franche-Comté, AgroSup Dijon, PAM UMR A 02.102, F-21000 Dijon, France; fiona.zoz@mxns.com (F.Z.); cosette.grandvalet@agrosupdijon.fr (C.G.); melanie.ragon@agrosupdijon.fr (M.R.); sebastien.dupont@agrosupdijon.fr (S.D.); laurent.beney@agrosupdijon.fr (L.B.); 2 Mérieux NutriSciences–70 Mail Marcel Cachin, F-38600 Fontaine, France; 3 ICB UMR CNRS 6303, Université de Bourgogne Franche-Comté, F-21078 Dijon, France; eric.lesniewska@u-bourgogne.fr; 4 Laboratory for Food Safety, French Agency for Food, Environmental and Occupational Health & Safety (ANSES), Université Paris-Est, F-94700 Maisons-Alfort, France; olivier.firmesse@anses.fr (O.F.); brigitte.carpentier@anses.fr (B.C.)

**Keywords:** *Listeria monocytogenes*, dehydration, rehydration, membrane permeability, envelope integrity, cultivability, surface

## Abstract

Although relative air humidity (RH) strongly influences microbial survival, its use for fighting surface pathogens in the food industry has been inadequately considered. We asked whether RH control could destroy *Listeria monocytogenes* EGDe by envelope damage. The impact of dehydration in phosphate-buffered saline (PBS) at 75%, 68%, 43% and 11% RH on the bacterial envelope was investigated using flow cytometry and atomic force microscopy. Changes after rehydration in the protein secondary structure and peptidoglycan were investigated by infrared spectroscopy. Complementary cultivability measurements were performed by running dehydration–rehydration with combinations of NaCl (3–0.01%), distilled water, city water and PBS. The main results show that cell membrane permeability and cell envelope were greatly altered during dehydration in PBS at 68% RH followed by rapid rehydration. This damage led cells to recover only 67% of their initial volume after rehydration. Moreover, the most efficient way to destroy cells was dehydration and rehydration in city water. Our study indicates that rehydration of dried, sullied foods on surfaces may improve current cleaning procedures in the food industry.

## 1. Introduction

*Listeria monocytogenes* is a foodborne pathogenic bacterium that can cause listeriosis, which is a serious foodborne illness and a major public health concern [1,2]. Although sporadic cases are uncommon, the incidence rate is generally estimated at around three cases per million population per year and about 9% for infants [1,2,3]. In 2017, the case fatality in the European Union was 13.8% among 1633 confirmed cases with known outcome [4]. This bacterium is found in many environments and is frequently present in food processing environments, where it can persist for many years and may contaminate a large variety of foods, including cheese, ready-to-eat foods, meat and fish products, and new food vehicles like ice cream [5].

Numerous studies have detected the presence of *L. monocytogenes* in different areas of food industries that are in direct contact or have no contact with foods (e.g., pipes, soil and processing equipment, such as meat slicers, conveyor belts and knives) on which it can persist [6,7,8,9]. Most serotypes found on surfaces and foods are 1/2a, 1/2b, 1/2c, 4a and 4b, which are also frequently responsible for listeriosis cases [10]. Serotypes 4b and 1/2a are mainly found in milk and dairy products [11,12,13,14]. Different strategies are implemented to fight *L. monocytogenes* because it can persist via surface adhesion (e.g., polypropylene, stainless steel and biofilm) and by forming with other bacteria [6,15,16,17].

Within the food industry, *L. monocytogenes* is exposed to different environmental conditions (e.g., temperature, relative air humidity [RH]) and, thus, encounters different stresses because contamination can occur at any point in the production cycle, including via handlers or by cross-contamination of raw materials in processing plants [15]. Some bacteria can adapt to and persist in stressful conditions. To fight both persistence and introduction into food processing environments, highly recommended technical strategies include compartmentalization of the processing lines to avoid cross-contamination [18,19]. Cleaning and disinfection programs are performed with chemical agents (e.g., acidic, alkaline or enzymatic agents) and thermal methodologies [15,20]. While complementary alternative strategies, such as bacteriophage, quorum sensing inhibitors and essential oils, have been proposed, RH has been inadequately considered. We have previously shown that RH should be considered for improving surface hygiene because the cultivability of different strains of *L. monocytogenes* suspended in phosphate-buffered saline (PBS) decreased dramatically at the ambient RH of 68% [21]. Although resistance to desiccation was strain-dependent, it was uncorrelated with strain origin or virulence, and serotype 1/2b strains were classified as resistant [22]. In general, we have also shown that the sensitivity and resistance of pathogens such as *Cronobacter sakazakii*, *Salmonella enterica/typhimurium* and *L. monocytogenes* depend on several parameters: e.g., composition of the suspending medium, dehydration and rehydration kinetics, magnitude of water activity, variation of the biological sample during dehydration and holding time at the specified RH [21,23,24,25]. However, the cellular mechanisms involved in pathogen dehydration–rehydration resistance remain poorly understood. Herein, we investigated the role of the plasma membrane and cell wall, which are the first cell structures to be altered because dehydration induces an osmotic stress and subsequent mechanical stress due to the loss of intracellular water. *L. monocytogenes* strains EGDe and SLCC2755 exhibit similar resistance to desiccation [22], whereas SLCC2755 displays a nonsense mutation in the *lmo2768* gene (encoding a potential permease ABC transporter with unknown function [26]), which could play a role in responses to desiccation and osmotic stresses [27] and in cell wall homeostasis [28]. A complementary study also showed that the plasma membrane plays a main role in pathogen inactivation during drying, as cell death was closely related to membrane permeabilization in *C. sakazakii* and *S. enterica* dried in milk powder [29].

Herein, to investigate the involvement of the cell wall and plasma membrane of *L. monocytogenes* EGDe in resistance to rehydration/rehydration, cells were suspended in PBS on polypropylene coupons and then dried for 3 h at 75%, 68% or 43% RH. Plasma membrane integrity was evaluated following the subsequent rapid rehydration via flow cytometry combined with propidium iodide (PI) fluorescent probe. Consistent with this, Fourier-transform infrared spectroscopy (FTIR) was used to assess changes in membrane fluidity and peptidoglycan structure. This method was also used to investigate the impact of dehydration–rehydration on the protein secondary structure. To better characterize the cell wall during drying (i.e., in the absence of rehydration), atomic force microscopy (AFM) was used to observe the morphology of dehydrated bacteria (from 99–43% RH) and to measure the variations in cell volume and cell surface elasticity/rigidity. Complementary observations and measurements were carried out after dehydration at 68% or 11% RH, followed by rapid rehydration in PBS. The main results showed that the cell envelope was dramatically altered after dehydration at 68% RH followed by rapid rehydration. Finally, survival was measured after a dehydration (75% RH for 3 h)–rehydration cycle of cells suspended in binary saline solutions (ranging from 3–0.01%), distilled water, Dijon city water or PBS. Subsequent rapid rehydration was carried out either with PBS or distilled water, or the same liquid matrix used for dehydration. The use of city water to suspend, dehydrate and rehydrate cells was the most efficient way to kill *L. monocytogenes* EGDe, certainly due to the presence of chlorine. Thus, this study highlighted RH management and the use of city water to fight *L. monocytogenes* in the food industry.

## 2. Materials and Methods

### 2.1. Bacterial Strain and Growth Conditions

The *L. monocytogenes* EGDe (animal isolate, serotype 1/a) strain used herein was supplied by the French National Agency for Food, Environmental and Occupational Health Safety (ANSES, Maisons-Alfort, France). The strain was stored on tryptic soy broth (TSB) supplemented with 20% glycerol (Sigma-Aldrich, St. Quentin Fallavier, France) at −80 °C. For revitalization, bacteria were isolated with a sterile swab on tryptic soy agar (TSA, Sigma-Aldrich) medium, incubated at 25 °C for 48 h and stored at 4 °C for a maximum of 1 month. Subculture was performed by inoculation of five colonies in 10 mL of TSB and incubated overnight at 25 °C. Final culture was performed by diluting the subculture to adjust the final OD_600_ to 0.005 in 50 mL of fresh TSB before incubation at 25 °C for 24 h to reach the stationary growth phase.

### 2.2. Preparation of Environmental Drying Chambers

Controlled environment drying chambers were hermetic plastic boxes (20 cm × 13 cm × 6 cm) containing a saturated salt solution to control RH [30]. A saturated solution of NaCl, KI, K_2_CO_3_ and LiCl (Sigma-Aldrich) was added to the bottom of the drying chambers to obtain 75%, 68% or 43% RH in the hermetic box. A ventilator of maximum 4.1 W with diameter 80 mm and height 25 mm with seven blades (Sunon; Radiospares, Beauvais, France) was placed in the drying chamber to control the rate of drying.

### 2.3. Desiccation and Rehydration Bacteria Processes

A stationary growth phase culture of EGDe (25 mL) was centrifuged for 10 min at 3645× *g* and washed once with PBS (Sigma-Aldrich). The pellet was suspended in 20 mL of PBS. For desiccation experiments, 10 μL of cell suspension with a final concentration of 1 × 10^9^ colony-forming units (CFU)/mL was placed on the hydrophobic polypropylene coupons (Scientix, Fougères, France) for flow cytometry analysis or coverslips coated with poly-d-lysine (Corning^®^ BioCoat™; Sigma-Aldrich) for AFM analysis. The samples were placed in the drying chamber on a rack to keep them above the salt solutions. The various conditions (43%, 68% and 75% RH for 3 h) were tested while the chambers were maintained at 25 °C. A Sunon (Radiospares) ventilator was placed in the drying chamber to increase mass transfer.

For instantaneous rehydration, 1 mL of PBS was deposited on the dried cells, which were then detached from the polypropylene coupon by 30 successive aspirating and dispensing cycles using a micropipette. For gradual rehydration, the dried cell samples were transferred to a closed chamber with 99% RH for 60 min at 25 °C. One milliliter of PBS was then deposited on the sample and bacteria were resuspended by 30 successive aspirating and dispensing cycles using a micropipette.

### 2.4. Measurement of Cell Cultivability

Bacteria viability was estimated by a spread plating method. After rehydration, cell suspensions were serially diluted, and appropriate 10-fold dilutions were plated on TSA. Colonies were counted after incubation for 48 h at 25 °C and recorded as CFU/mL. Bacterial proportion was defined as the percentage ratio of TSA counts before and after desiccation.

### 2.5. Determination of Changes in Cell Component Structure by FTIR

Changes in membrane fluidity, protein and cell wall structure were estimated using transmission FTIR spectroscopy. The spectra were measured using dehydrated samples at 25 °C and 75%, 68% and 43% RH for 3 or 16 h. Controls were maintained at 99% RH and 25 °C. Cells were dehydrated on a ZnSe crystal instead of a polypropylene coupon, as described in Section 2.3, to avoid any transfer of dried bacteria from a coupon to a ZnSe crystal. One milliliter of a cell suspension with a concentration of 10^9^ cells/mL^−1^ in PBS was centrifuged three times at 5100× *g* for 10 min at 25 °C to remove the supernatant, which can induce signal noise. The cell pellet was spread at the center of the ZnSe crystal previously placed in the drying chambers on a rack to keep it above the salt solutions, as described in Section 2.3. To analyze the samples, the ZnSe crystal was removed from the drying chamber and then a second ZnSe crystal was used to sandwich the cells. To avoid any release of cells, the periphery of the sandwich was wrapped with a parafilm Table Such a sandwich was necessary because the FTIR spectrometer was not in a biosafety level 2 laboratory. Samples were placed in the FTIR spectrometer (Vector 22; Bruker, Karlsruhe, Germany), which was equipped with a mercury–cadmium–telluride (MCT) detector. The spectral resolution was 4 cm^−1^. To obtain the spectra for each sample, 50 scans were recorded from 900 cm^−1^ to 4000 cm^−1^ and analyzed using OPUS 6.5 software (Bruker). Several data preprocessing algorithms were used to analyze all FTIR spectra. Data preprocessing first included a smoothing of the spectrum (17 smoothing points, provided by OPUS 6.5 software) and then a second derivative transformation. To evaluate protein secondary structure or the intensity variation of C–O–C/P–O–C peak and membrane fluidity, the second derivative spectra were normalized with respect to the corresponding tyrosine band (around 1515 cm^−1^) or the υCH_3_ symmetric stretching band (around 2870 cm^−1^).

### 2.6. Determination of Cell Integrity by Flow Cytometry

Membrane integrity after desiccation and gradual or instantaneous rehydration was determined by flow cytometry analysis using the fluorescent probe PI (Molecular Probes, Invitrogen, France). PI penetrates cells only when the cell envelope is permeabilized and/or altered. Nondried stained cells and cells treated at 90 °C for 45 min and subsequently stained with PI were used as controls for PI negative and positive histogram regions, respectively. Control, heat-treated (90 °C, 45 min) or dried cells subsequently rehydrated progressively or instantaneously were adjusted to a concentration of 10^7^ cells/mL, 1 μg/mL PI was added, and samples were incubated for 10 min at 25 °C in the dark.

Analysis of *L. monocytogenes* was performed on an Accuri C6 flow cytometer (Becton Dickinson, San José, CA, USA) with a laser excitation at 488 nm. Red fluorescent of cells stained with PI was collected in the FL3 channel (>670 nm). For each sample, 10,000 events were collected at an average speed of 300 events/s. All data were collected in at least four independent experiments and analyzed with CSampler software (Becton Dickinson).

### 2.7. Observation of L. monocytogenes by AFM

Images of cells dried or instantaneously rehydrated onto coverslips coated with poly-d-lysine were collected in the air or in liquid PBS for control (cells incubated to 99% RH) using a Multimode NanoScope 8 microscope (Bruker, Veeco Instruments, Santa Barbara, CA, USA). A ScanAsyst-HR-AIR cantilever (Bruker) of spring constant k = 0.4 N/m was used for measurement in peak force mode and the scan rate was 1 Hz. Representative AFM images of cells before and after desiccation and rehydration were derived from at least three experiments. Images were analyzed with NanoScope Analysis 1.5 software (Bruker).

### 2.8. Measurement of Bacterial Cell Envelope Elasticity by AFM in Force Volume Mode and Young’s Modulus

Force mode volume allowed image acquisition with 128 force curves line-by-line. These curves allow measurement of sample surface elastic behavior; i.e., the elasticity of *L. monocytogenes*’ surface after desiccation.

Young’s modulus was calculated using the proposed Hertz model

(1) $$E=\frac{3\left(1-v^{2}\right)K}{4\sqrt{R}}$$

where *E* is the Young’s modulus, *v* is the Poisson ratio, *K* (N·m^−1^) is the spring constant of the AFM cantilever and *R* is the radius of the sphere of the AFM cantilever.

Force volume measures were collected in peak force mode with a ScanAsyst-HR-AIR cantilever of spring constant *K* = 0.4 N·m^−1^. Force volume measures were repeated at least three times in independent experiments.

### 2.9. Statistical Analysis

All data were collected in four independent experiments. To compare the various results obtained herein, the variance homogeneity (F-test) was assessed. Our results revealed a homogeneous variance (*p* > 0.05). ANOVA and Tukey’s honestly significant difference test (with *p* < 0.05 indicating statistical significance) were performed to determine whether significant differences existed between different stress conditions. Analyses were performed using R software (version 3.1.2).

## 3. Results and Discussion

### 3.1. Impact of Drying and Rehydration in PBS on Plasma Membrane Permeability

**Figure 1:**
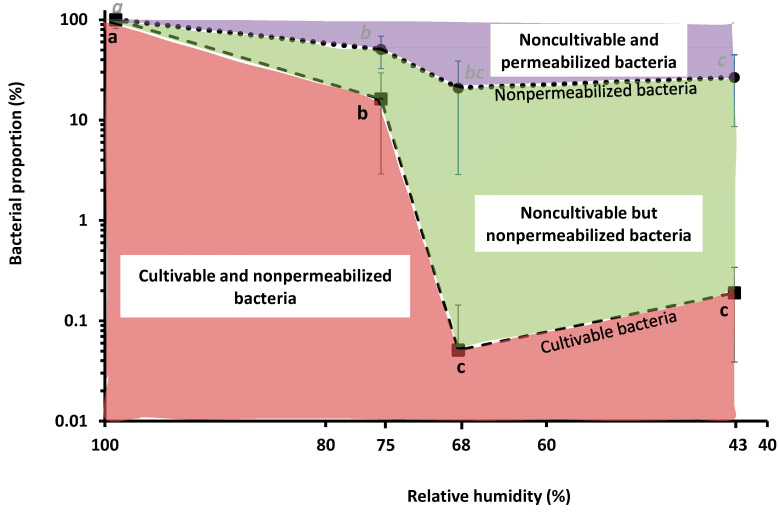
Impact of drying in PBS on *L. monocytogenes* EGDe cultivability and plasma membrane permeability. Bacteria were dried at 75%, 68% or 43% RH for 3 h at 25 °C, then instantaneously rehydrated in PBS. Undried bacteria were maintained at 99% RH (control). (**- - -**) represents the proportion of cultivable bacteria and (• • •) the proportion of nonpermeabilized bacteria. The proportion of cultivable bacteria was estimated using the CFU counting method, the proportion of nonpermeabilized bacteria was estimated by labelling cells with PI and using a flow cytometer. Letters indicate significant differences (*p* < 0.05) based on Tukey post hoc tests. Two independent Tukey tests were run: one for cultivability measurement (black letters) and another for nonpermeabilized bacteria counting (italics grey letters).

We previously showed that *L. monocytogenes* strains EGDe, CCL500, CCL128 and LO28 suspended in PBS were more sensitive to dehydration at 68% RH than at 75%, 43% or 11% when followed by rapid rehydration in PBS [21]. This experiment was repeated with EGDe using bacteria suspended in PBS, dried to 75%, 68% or 43% RH for 3 h at 25 °C and then instantaneously rehydrated in PBS before counting CFUs and (non-)membrane permeabilized bacteria (Figure 1). Consistent with our previous results, drying at 68% and 43% induced a significant decrease in CFU counts to about 0.1% (about −3 log reduction) [21]. Moreover, at the same RH levels, the proportion of nonpermeabilized bacteria decreased to about 20%. Thus, plasma membrane permeabilization played a significant role in cell death at 68% and 43% RH because >70% of bacteria were permeabilized. This is also consistent with previous studies showing that permeabilization of the plasma membrane occurred during a dehydration–rehydration cycle in bacteria and yeast [29,31].

The difference between the proportion of nonpermeabilized and cultivable bacteria led us to estimate the proportion of noncultivable but nonpermeabilized bacteria. About 20% of such bacteria were present at 68% and 43% RH, meaning that bacterial subpopulations composed the whole population. Such phenotypic heterogeneity may be partly explained by intrinsic (originates from the stochastic nature of gene expression) and extrinsic (linked to the metabolic state of cells) cell heterogeneity [32]. Biological heterogeneity could also be related to plasma membrane physiology because three main subpopulations, each characterized by a specific membrane phenotype (related to permeability and potential), were found in stationary growth phase Escherichia coli cells [33].

### 3.2. Impact of Drying and Rehydration in PBS on the Cell Envelope

The dried cell components were analyzed using FTIR and AFM. FTIR was used to evaluate changes in plasma membrane fluidity, protein secondary structure and cell wall composition. Complementarily, AFM was used to observe changes in cell envelope morphology (as the envelope breaks), to measure its elasticity and to measure cell volume variations. Both methods have the advantage of not needing to study fully hydrated cells. Thus, it is possible to study and characterize cells at intermediate dehydration levels.

**Figure 2:**
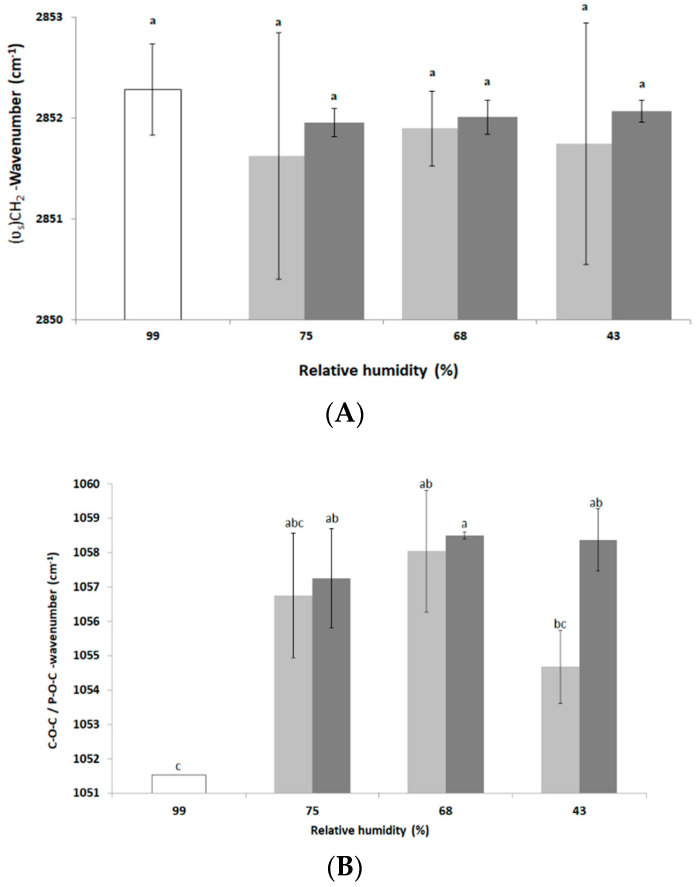
FTIR transmission spectroscopic analysis of *L. monocytogenes* EGDe components after dehydration. Cells were dehydrated at 25 °C and 75%, 68% or 43% RH for 3 h (light grey bars) or 16 h (dark grey bars) and then instantaneously rehydrated in PBS. Untreated cells (control, white bars) were maintained at 99% RH. FTIR spectroscopic analysis was performed on the second derivative spectra as explained in the Materials and Methods section. Changes in frequency of (**A**) symmetrical (υ_s_)CH_2_ and (**B**) C–O–C/P–O–C peaks. Error bars correspond to the standard deviation calculated from at least three independent experiments (except for control in (**B**) for which only one repetition could be run). Histogram bars followed by different letters indicate significant differences (*p* < 0.05) as determined by the Tukey post hoc test.

During environmental stress, the fluidity of the plasma membrane can be modified in two ways. First, in the context of moderate osmotic challenges in nutritive media, microorganisms modify their membrane fluidity, leading to homeoviscous regulation of the membrane [34]. Such regulation involves chemical changes allowing maintenance of the membrane fluidity. Second, in the context of severe osmotic challenges, the decrease in free water leads to a decrease in water–phospholipid polar head interaction and then to a decrease in membrane fluidity and phase changes. For example, the yeast *Saccharomyces cerevisiae* and the bacterium *E. coli* decrease their membrane fluidity when suspended in a hyperosmotic medium [35,36]. Using FTIR spectroscopy, changes in the membrane fluidity of *L. monocytogenes* were determined by measuring the changes in the vibrational modes of the wavenumbers of the symmetric (υ_s_)CH_2_ stretching bands of the phospholipids located at around 2850 cm^−1^ [35,37]. The results presented in Figure 2A show that no change in the frequency of symmetrical (υ_s_)CH_2_ and, thus, in plasma membrane fluidity occurred after drying for 3 h and 16 h. Nevertheless, after 3 h at 75% and 43% RH the signal was unstable, based on the large standard deviations disallowing variation in plasma membrane fluidity.

This might also be explained biologically by membrane reorganization phenomena producing an instability (as membrane vesiculation) of this cell structure during drying [35,38]. After 16 h of drying, the standard deviations are somewhat lower than those of the control and the frequency of vibration of the symmetrical (υ_s_)CH_2_ during different drying conditions tends to decrease compared to the control. This could reveal a more structured organization of the phospholipids of the plasma membrane [35]. To assess the impact of dehydration on the cell wall structure of *L. monocytogenes* EGDe, changes in the wavenumber of the C–O–C and P–O–C symmetrical bonds of the peptidoglycan were measured after 3 h and 16 h at 75%, 68% and 43% RH. The results presented in Figure 2B show that vibration frequencies of the C–O–C and P–O–C symmetrical bonds tended to increase at the three drying conditions compared to the control (maintained at 99% RH), which may indicate an alteration in the peptidoglycan of the cell wall [39]. Such an alteration, indicative of a decrease in the amount of polysaccharides at the cell wall, could be related to adaptive mechanisms as previously shown in a cobalt-acclimated *Bacillus* sp. group [39]. As previously reported, bacteria are able to “fine tune” peptidoglycan properties (as surface density and degree of cross-linkage) to adapt to changing environmental conditions [40]. However, the advantages of such changes are still not well understood. Unfortunately, changes in C–O–C and P–O–C peak cannot be used to predict cell cultivability since, after dehydration for 3 h, cell cultivability was significantly different between 75% and 68% RH (Figure 1) whereas the infrared signal was similar in both conditions (Figure 2B).

**Figure 3:**
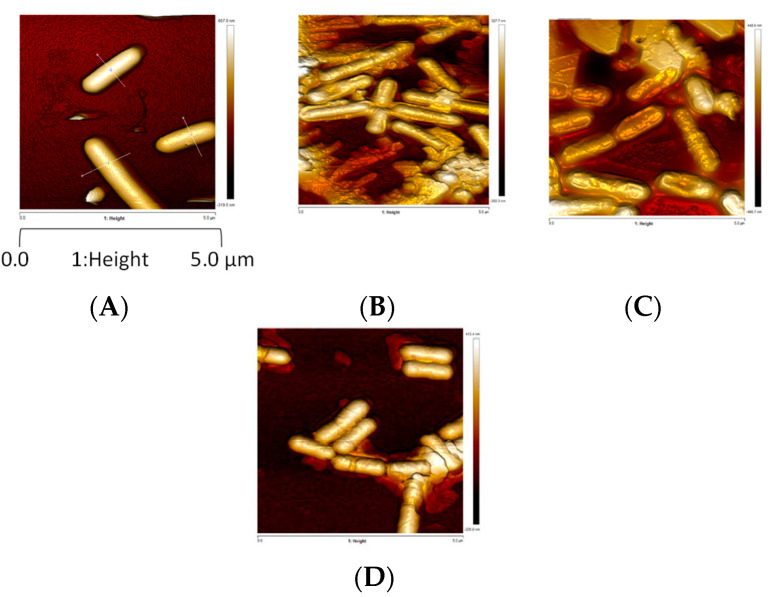
*L. monocytogenes* EGDe morphology observed by AFM height images after dehydration. Z-scale approx. 250 nm. Cells were (**A**) hydrated in PBS at 99% dehydrated or dehydrated in PBS at (**B**) 75%, (**C**) 68% or (**D**) 43% RH for 3 h at 25 °C. Cells were immobilized on a glass slide coated with poly-d-lysine.

AFM was also used to complimentarily assess the impact of drying and rehydration on *L. monocytogenes* envelope topology, morphology and mechanical properties. AFM, a powerful, noninvasive tool, has been widely used to analyze surface physical properties of food microorganisms [41]. The AFM images shown in Figure 3 indicate that bacteria hydrated at 99% RH for 3 h, and those dehydrated at 75% and 43%, exhibit a smooth, well-structured surface. However, dehydration at 68% RH for 3 h induced severe changes in surface morphology: they are flat with a distorted surface, as confirmed by the surface profiles (Table 1). Dehydrated cells at 68% RH exhibiting a distorted surface could be unable to repair peptidoglycan structure, meaning that peptidoglycan turnover was inhibited. Any irreversible alteration of the peptidoglycan, and thus the cell wall, could affect the main mechanical properties, such as the maintenance of the cell shape, by assuming surface-tension forces and the turgor resistance [42]. Thus, inhibition of the peptidoglycan turnover could affect *L. monocytogenes* survival [43]. Intermediate RH (68%) appeared to be the most detrimental for surface morphology, and so for peptidoglycan structure. Peptidoglycan’s behavior is typical of glassy polymer with characteristics of a polymer above its glass transition at high levels of humidity [42]. Its mechanical properties (strength, stiffness and elasticity) that depend on several factors (such as RH and the presence of ions) are maintained up to about 50% RH in *Bacillus subtilis* [42]. Dramatically, changes observed at 68% RH may be due to the combination of changes in peptidoglycan physico-chemical properties and the decrease (but not a cancellation) in the molecular mobility due to water removal [43]. RH lower than 68% could slow down the molecular mobility, and, thus, many biochemical reactions (i.e., drying of microorganisms of interest).

Cell volumes of dehydrated bacteria were one third of those hydrated, which led to surface excess around 50% (Table 1). The decrease in volume could be explained by the hyperosmotic stress (inherently occurring during solute concentration in the suspending medium due to water evaporation) and, thus, osmotic water release from the inner cell. The decrease in cell volume and cell shrinkage due to hyperosmotic pressure increase have been previously suggested in *E. coli* by means of AFM [44]. Interestingly, Young’s modulus, which reflects cell surface elasticity/rigidity, dramatically increased at 75% RH, then decreased at 68% and 43% RH (Table 1). The lower the Young’s modulus, the greater the elasticity, meaning that at 68% and 43% RH, the cell surface elasticity increased. Essentially, the increase in elasticity could be explained by the partial loss of intracellular water leading to a more elastic/flexible interphase with the surrounding medium [44]. It may be that increases in the elasticity/flexibility of this interface could also be related to a significant decrease in turgor pressure. Consistent with this, a previous study showed that the cell wall peptidoglycan in Group B *Streptococcus* was less stretched and under lower tensile stress in physiological osmolarity than in the hypotonic medium [45]. Moreover, changes in the surface elasticity could be related to cell wall expansion because the isolated walls of *Bacillus megaterium* display contraction and then expansion when osmotic pressure is increased in the presence of NaCl [46]. Thus, AFM led us to confirm that, during drying, bacteria were exposed to a hyperosmotic stress leading to transmembrane water flows from the inner to outer cells. Such water flows could explain bacterial envelope hardening during the initial stage of drying. However, envelope relaxation and solidification, respectively, occurred if the cells were dried at 68% or 43% RH.

**Figure 4:**
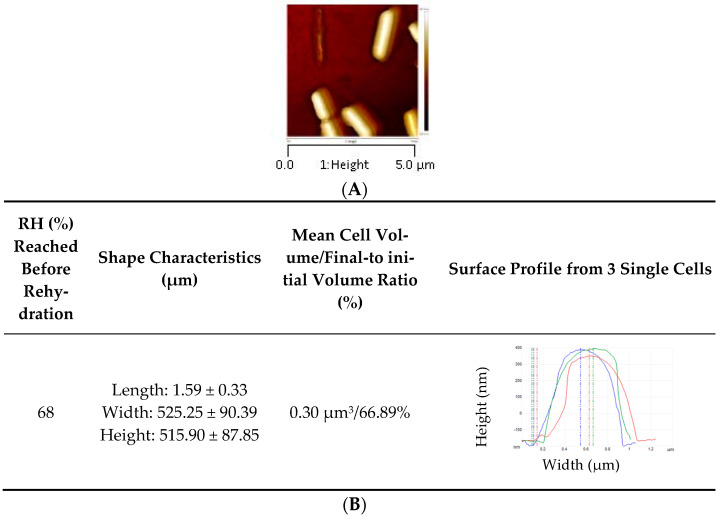
*L. monocytogenes* EGDe morphology observed by AFM after dehydration and rapid rehydration in PBS. Cells were dehydrated in PBS at 68% RH for 3 h at 25 °C. AFM height images (with a Z-scale approx. 250 nm) of cells dehydrated at (**A**) 68% RH followed by rapid rehydration. (**B**) *L. monocytogenes* EGDe morphology characteristics. Cells were immobilized on a glass slide coated with poly-d-lysine.

We previously showed that a subsequent instantaneous rehydration played a main role in *L. monocytogenes* death [21,23]. Here, we observed the cell morphology of bacteria rehydrated after drying at 68% RH (Figure 4). This indicates that a massive water entry into the cell occurred during rehydration. However, when the cells were previously dehydrated at 68% RH, they recovered only 66.89% of their initial volume after subsequent rehydration, meaning that plasma membrane permeabilization occurred. This is consistent with the results presented in Figure 1 showing that about 70% of cells were permeabilized at 68% RH. Plasma membrane permeabilization certainly occurred during instantaneous rehydration, as was previously shown in the yeast *S. cerevisiae* [30]. Moreover, instantaneous rehydration induced plasma membrane permeabilization and vesicle formation in *E. coli* and *S. cerevisiae* cells dehydrated in a liquid medium by adding glycerol [31,47,48].

### 3.3. Impact of Drying and Rehydration in PBS on Protein Secondary Structure

**Figure 5:**
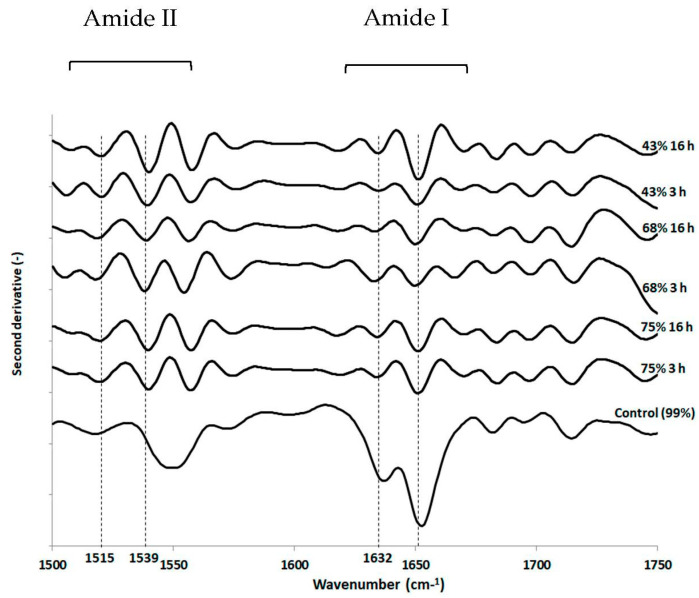
FTIR transmission spectroscopic analysis of *L. monocytogenes* EGDe components after dehydration. Cells were dehydrated at 25 °C and 75%, 68% or 43% RH for 3 h or 16 h and then instantaneously rehydrated in PBS. Untreated cells (control) were maintained at 99% RH. FTIR spectroscopic analysis was performed on the second derivative spectra, as explained in the Materials and Methods section. Randomly selected second derivative spectra ranging from 1500 cm^−1^–1750 cm^−1^ in the Amide I and Amide II regions are presented.

The protein secondary structure was also analyzed using FTIR spectroscopy (Figure 5). Unexpectedly, regardless of RH/time pairings, no significant changes were observed in the amide I band (1600–1650 cm^−1^) between the different drying conditions and the control. This observation was based on principal component analyses of processed spectra (not shown) in the range 1500–1750 cm^−1^ and on multiple comparisons of *α*-helix (around 1650 cm^−1^) to *β*-sheet (around 1632 cm^−1^) peak height ratio of processed spectra with ANOVA and Tukey tests (not shown). Although protein secondary structure seemed well preserved during dehydration, opposite observations have been reported, such as the changes induced during dehydration in the protein secondary structure of yeast cells analyzed by ATR-FTIR [49]. Nevertheless, we observed changes in the amide II band (1500–1550 cm^−1^) in dehydrated cells in comparison with hydrated cells (99% RH). Indeed, a more pronounced well-known peak appeared in dehydrated cells around 1539 cm^−1^ meaning that cell death could be partly related to changes in the amide N-H deformation and C-N stretch, and the bending modes of CH_3_ and CH_2_ groups present in all biomolecules. Moreover, it should be noted that the recorded signal intensity may have been limited because the cells were sandwiched between ZnSe crystals.

### 3.4. Impacts of Drying and Rehydration on L. monocytogenes in the Food Industry Context

**Figure 6:**
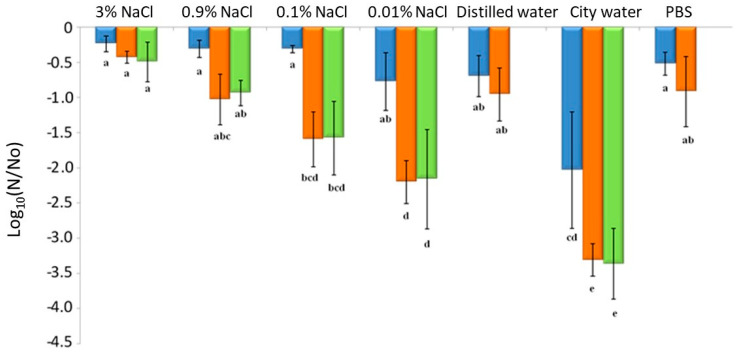
The decreased cultivability of *L. monocytogenes* strain EGDe dehydrated at 25 °C and 75% RH for 3 h and rehydrated in various liquid matrices. The bacteria were suspended and then dried in different solutions: 3% NaCl (0.989 a_w_), 0.9% NaCl (0.997 a_w_), 0.1% NaCl (0.999 a_w_), 0.01% NaCl (0.999 a_w_), distilled water (1.000 a_w_), city water (1.000 a_w_) and PBS (1.000 a_w_). Subsequently, rapid rehydration was performed in (■) PBS, (■) distilled water or (■) the same liquid matrix used for dehydration. Error bars correspond to the standard deviation calculated from four independently repeated experiments. Histogram bars followed by different letters indicate significant differences (*p* < 0.05) determined by Tukey post hoc tests.

Although PBS is commonly used in the laboratory as a model-suspending medium, it is not used in the food industry context. To better mimic conditions encountered by bacteria in the food industry, different protocols were tested with NaCl, the most widely used food preservative and a common foodstuff component. Herein, to manage cell cultivability, the well characterized EGDe was dried to 75% RH for 3 h at 25 °C in various liquid matrices with different a_w_: 3% NaCl (0.989 a_w_), 0.9% NaCl (0.997 a_w_), 0.1% NaCl (0.999 a_w_), 0.01% NaCl (0.999 a_w_), distilled water (1.000 a_w_), Dijon city water (1.000 a_w_) and PBS (1.000 a_w_, composed of different salts including 0.8% NaCl). Subsequent rapid rehydration was carried out with PBS, distilled water or the same liquid matrix used for dehydration. Here, NaCl concentrations were chosen based on those found in some foodstuffs (Table 2) that may eventually lead to contaminated food sullying factory surfaces (e.g., floors, walls, machines). Cells were dehydrated at 75% RH instead of 68% RH to test the possibility of improving the process in the context of the food industry, and in terms of cell killing while minimizing the energy cost linked to the dehydration step. In accordance with previous studies [50,51,52], the results presented in Figure 6 show that high salt concentration (3% and 0.9%) protected cells from the dehydration–rehydration cycle.

Several hypotheses might explain this protective effect. In the presence of NaCl, cells dry less quickly, remain in an aqueous solution longer and have less contact with the oxygen in the air compared with cells dried at low NaCl concentrations. High NaCl concentrations can also induce osmotic stress responses such as the accumulation or synthesis of stress proteins. The MrpABCDEFG Na^+^/H^+^ carrier plays an important role in the osmoadaptation of *L. monocytogenes* and the Na^+^ ion is involved in the mechanisms of resistance to stress, which make it possible to balance the osmotic pressures between the intracellular and extracellular medium [53].

Rehydration with a PBS solution resulted in higher survival after drying than using low concentrations of salt and city waters. The presence of a higher salt concentration in PBS (0.8% NaCl) could also minimize the hypoosmotic shock caused by rehydration [45]. This decreases the quantity of water entering cells during rehydration. Thus, the cell volume variation and mechanical constraints on the plasma membrane may be reduced in this condition. Drying in city water is the most lethal cell treatment. However, water from the city network contains many compounds (conductivity 442–526 μS/cm) including calcium (100.89 mg/L), sulfate (15.2 to 36 mg/L), sodium (4.7 mg/L), chlorides (8.5–12.4 mg/L), potassium (1.53 mg/L), nitrates (3.4–21.5 mg/L), ammonium (<0.01 mg/L), traces of pesticides (<0.02 μg/L) and disinfection by-products that can have harmful effects on cells regardless of drying. Chlorine compounds are widely used as sanitizing agents in the food industry and drinkable water treatments. Several studies have shown the inactivation effects of such chlorine compounds on *L. monocytogenes* [54,55]. The lethal effect could be explained by membrane damage leading to a loss of permeability control [56,57]. The mechanisms inducing changes in membrane permeability remain unclear but may be linked to the alteration (probably oxidation) of proteins and lipids [56,57,58,59]. Moreover, rehydration can induce transitory membrane permeabilization, allowing compounds of the rehydration solution to enter the cells [60]. This phenomenon could explain the drastic effect of rehydration with a solution containing harmful molecules. Rehydration with city network water was used to replicate the disinfection protocols carried out on production sites as closely as possible. However, this water is unstandardized and its composition (i.e., chlorine concentration) depends on the water network.

## 4. Conclusions

Surface decontamination in the food industry by means of RH management appears to be a promising approach that could be used in combination with current cleaning and disinfection procedures. The EcoSec research project (funded by the French National Research Agency and that involved one food company and one company specialized in dry air technology and its industrial applications for drying and dehumidification) allowed us to note that the RH of food processing environments, and more particularly 68% RH, could be managed using a desiccant wheel. Herein, we showed that dehydration at 68% RH followed by rapid rehydration alters the structure of the *L. monocytogenes* EGDe envelope. Obviously, applying rehydration is moot in a food industry decontamination context. However, the best results in terms of bacterial destruction were clearly obtained when samples were rehydrated with city water (>3 log reduction), which is used for cleaning in the food industry. Thus, using city water to rehydrate contaminated surfaces on which sullied foods have had time to dry is possible as it can be incorporated as a first step in cleaning procedures. Nevertheless, future work should be performed to better understand the role of the suspending medium in bacterial resistance or death during dehydration and rehydration. Certainly, the media of interest for the food industry are those that compose food matrices that can sully surfaces (e.g., fish and meat juices).

## Figures and Tables

**Table 1:** *L. monocytogenes* EGDe morphology characteristics observed by AFM images in hydrated and dehydrated states as specified in Figure 3.

RH (%)	Shape Characteristics (µm)	Mean Cell Volume/Final-to Initial Volume Ratio (%)	Mean Surface/Surface Excess (%)	Surface Profile from 3 Single Cells	Young Modulus (MPa)
99	Length: 1.78 ± 0.33 Width: 653.08 ± 121.27 Height: 555.56 ± 18.48	0.45 µm^3^/100%	3.38 µm^2^/0.00%	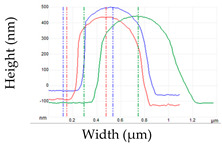	2.48 ± 0.57 ^c^
75	Length: 1.38 ± 0.47 Width: 433.09 ± 76.24 Height: 375.30 ± 32.60	0.15 µm^3^/35.28%	1.75 µm^2^/48.15%	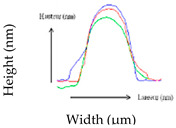	152.63 ± 11.54 ^a^
68	Length: 1.42 ± 0.37 Width: 495.27 ± 67.13 Height: 323.38 ± 59.14	0.16 µm^3^/37.50%	1.83 µm^2^/45.70%	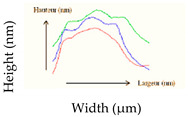	41. 58 ± 16.10 ^b^
43	Length: 1.32 ± 0.12 Width: 409.50 ± 31.94 Height: 382.16 ± 7.98	0.14 µm^3^/32.31%	1.64 µm^2^/51.38%	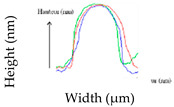	26. 73 ± 14.86 ^bc^

Young’s modulus values with different letters indicate significant differences (*p* < 0.05) as determined by Tukey post hoc tests.

**Table 2:** NaCl concentration in some foodstuffs found in the French food composition table.

Food Item	Average NaCl Content (g/100 g)	Confidence Code *
Salty snacks, crackers, reduced fat	3	A
Braised ham on the bone	2.97	C
American-style sauce, prepacked	2.94	A
Lardoons, raw	2.72	D
Ketchup, prepacked	2.59	A
Salad dressing, reduced fat, prepacked	2.57	A
Atlantic herring, marinated, or rollmops	2.56	C
Caribbean-style fish fritters, fish acras	1.24	A
Tabbouleh, prepacked	1	A
Seafood, cooked, frozen	0.99	C
Marine fish, white, without skin, cooked	0.98	D
Mackerel, smoked	0.96	C
Vegetable dish for baby, w meat/fish and starch, from 6–8 months	0.2	A
European whitefish, raw	0.13	B
Carrot juice, pure juice	0.1	A
Pork, roast, raw	0.1	A
Yogurt, ewe’s milk, wholemilk, plain	0.1	A
Jam, strawberry or cherry	0.018	A
Fruit juice, from concentrate (average)	0.012	B
Fruit juice, mixed, juice and fruit puree	0.01	A

The data are from ANSES. Table de composition nutritionnelle des aliments Ciqual 2020. (https://ciqual.anses.fr, accessed 22 April 2021). * To inform users about the data quality, the ANSES–CIQUAL food composition table provides a confidence code for each value. This code characterizes the quality of the average content value, ranging from A (very reliable) to D (less reliable). The reliability of the published data is generally estimated according to their representation in the French market, recentness and analytic method.

## Data Availability

Data are contained in the article.
